# Dosing Regimen of Enrofloxacin Impacts Intestinal Pharmacokinetics and the Fecal Microbiota in Steers

**DOI:** 10.3389/fmicb.2018.02190

**Published:** 2018-09-19

**Authors:** Kaitlyn M. Ferguson, Megan E. Jacob, Casey M. Theriot, Benjamin J. Callahan, Timo Prange, Mark G. Papich, Derek M. Foster

**Affiliations:** ^1^Department of Population Health and Pathobiology, College of Veterinary Medicine, NC State University, Raleigh, NC, United States; ^2^Department of Clinical Sciences, College of Veterinary Medicine, NC State University, Raleigh, NC, United States; ^3^Department of Molecular and Biomedical Sciences, College of Veterinary Medicine, NC State University, Raleigh, NC, United States

**Keywords:** antimicrobial resistance, fluoroquinolone, cattle, microbiome, pharmacokinetics

## Abstract

**Objective:** The intestinal concentrations of antimicrobial drugs that select for resistance in fecal bacteria of cattle are poorly understood. Our objective was to associate active drug concentrations in the intestine of steers with changes in the resistance profile and composition of the fecal microbiome.

**Methods:** Steers were administered either a single dose (12.5 mg/kg) or 3 multiple doses (5 mg/kg) of enrofloxacin subcutaneously every 24 h. Enrofloxacin and ciprofloxacin concentrations in intestinal fluid were measured over 96 h, and the abundance and MIC of *E. coli* in culture and the composition of the fecal microbiota by 16S rRNA gene sequencing were assessed over 192 h after initial treatment.

**Results:** Active drug concentrations in the ileum and colon exceeded plasma and interstitial fluid concentrations, but were largely eliminated by 48 h after the last dose. The concentration of *E. coli* in the feces significantly decreased during peak drug concentrations, but returned to baseline by 96 h in both groups. The median MIC of *E. coli* isolates increased for 24 h in the single dose group, and for 48 h in the multiple dose group. The median MIC was higher in the multiple dose group when compared to the single dose group starting 12 h after the initial dose. The diversity of the fecal microbiota did not change in either treatment group, and taxa-specific changes were primarily seen in phyla commonly associated with the rumen.

**Conclusions:** Both dosing regimens of enrofloxacin achieve high concentrations in the intestinal lumen, and the rapid elimination mitigates long-term impacts on fecal *E. coli* resistance and the microbiota.

## Introduction

Some studies have demonstrated an association between antimicrobial administration and antimicrobial resistance (AMR) in fecal bacteria of cattle (Kanwar et al., 2013; Zaheer et al., 2013; Hog and Korsgaard, 2017). Yet others demonstrate little impact of antimicrobial therapy on AMR in fecal microbiota of cattle (Checkley et al., 2010; Morley et al., 2011; Schmidt et al., 2013). Methodological variation in identifying and characterizing antimicrobial resistance in the studies investigating AMR associated with treatment in feedlots prevents meaningful comparison of their results. Additionally, conclusions are often drawn from single bacterial isolates, selected resistance genes, or over varying sampling times, which further confounds interpretation and comparison between studies (Benedict et al., 2013, 2014).

Parenteral antibiotics administered to treat infections diffuse into the intestine and influence the population and susceptibility of the intestinal bacteria (Lindecrona et al., 2000; Ferran et al., 2013; Foster et al., 2016). However, there is little known about the duration of this effect, particularly on potential foodborne pathogens. Measurement of active, unbound drug concentrations in the intestinal lumen is necessary to correlate pharmacokinetic-pharmacodynamic (PK-PD) indices with microbial changes. Based on evidence that the exposure relationships are important for development of antimicrobial resistance (Martinez et al., 2012), it is possible that resistance can be affected by the dose and frequency of drug administration (Enne, 2010).

In the US, there are two approved dosing regimens for enrofloxacin for treating respiratory disease in cattle, but little is known about the relative impact of these dosing regimens on AMR. It is presumed that each dosing regimen has similar efficacy; therefore, impact of each regimen on AMR may provide a rational basis for choosing one regimen over the other. Further, understanding the relationships between dosing regimen of enrofloxacin and AMR is critical due to the importance of fluoroquinolones in human health. The objective of this study was to model the PK of the active concentrations of enrofloxacin and its metabolite, ciprofloxacin, in the intestine of steers, and correlate these PK indices with changes in the MIC of *E. coli* and fecal microbiome. Our hypothesis is that the single 12.5 mg/kg dose of enrofloxacin will achieve a greater maximum concentration of active drug within the intestine resulting in less selection for AMR bacteria and changes in the microbiota due to the brief drug exposure.

## Materials and methods

### Animals and treatments

This study was approved by the North Carolina State University Institutional Animal Care and Use Committee. Twelve healthy, 6-months-old Holstein steers (125 to 241 kg) were obtained as in previous studies (McKellar et al., 1999; TerHune et al., 2005; Davis et al., 2007; Foster et al., 2016). Sample size was determined based on previous PK-PD studies in cattle demonstrating differences in drug concentrations in different sampling locations (Davis et al., 2007; Foster et al., 2016). At 24–48 h post-probe placement (described below), steers received either a single subcutaneous injection of 12.5 mg/kg enrofloxacin (Baytril 100®; Bayer Animal Health, Shawnee Mission, KS) or three subcutaneous injections of 5.0 mg/kg enrofloxacin (Baytril 100®) administered once each day on 3 sequential days. Treatments were administered in a crossover design, with each calf receiving both treatments separated by a minimum washout period of 10 days, accounting for at least six drug half-lives from last dose. Half of the steers (*n* = 6) received the single dose regimen first, while the other half received the multiple dose regimen first.

### Plasma collection

Prior to drug administration, a jugular catheter (Intracath®, Becton Dickinson; Franklin Lakes, NJ) was inserted in the jugular vein. Blood samples were collected at time 0, 5, 10, 15, 30, 45, 60, 90 min and 2, 4, 6, 8, 24, 36, and 48 h after the single dose for optimum pharmacokinetic modeling and to encompass at least three drug half-lives, accounting for 90% of drug elimination from the plasma. In the multiple dose group, additional samples were collected at 2, 4, 6, 8, 12, and 24 h after the second dose, and 5, 10, 15, 30, 45, 60, 90 min and 2, 4, 6, 8, 24, 36, and 48 h after the third dose. The tubes were briefly stored on ice before centrifugation at 1,000 × *g* for 10 min to collect plasma and stored at −80°C until assayed.

### Interstitial fluid collection

An *in-vivo* ultrafiltration probe (UF-3-12, BAS; Bioanalytical Systems, West Lafayette, IN, USA) was inserted in the subcutaneous space above the shoulders as described previously (Davis et al., 2007; Messenger et al., 2012). The interstitial fluid (ISF) was collected at time 0 and appropriate intervals for each drug, which will account for approximately three drug half-lives post-administration. The collected fluid was immediately frozen at −80°C until assayed.

### Placement of intestinal ultrafiltration probes

Surgical procedures took place 24–48 h prior to enrofloxacin administration over 4 days with three surgeries per day as previously described (Warren et al., 2014) with the only significant modification being the use of standing-restraint and local anesthesia instead of general anesthesia. Local anesthesia was provided by infusion 15 ml of 2% lidocaine above and below the transverse processes of lumbar vertebrae L1, L2, L3, and L4 in order to anesthetize the dorsal and ventral spinal nerves T13, L1 and L2. Adequacy of anesthesia was determined by pricking the skin of the flank prior to the incision. Steers received 2.2 mg/kg of flunixin meglumine intravenously immediately prior to surgery and 24 h later. The collecting loops of an ultrafiltration probe (UF-3-12, BAS; Bioanalytical Systems, West Lafayette, IN, USA) were inserted into the lumen of the ileum and spiral colon. The free ends of the probes were exteriorized through the abdominal wall. To collect the ultrafiltrate, a 3 mL evacuated tube with no additive (Vacutainer®, Becton-Dickinson) was inserted onto the needle of the vacuum vial needle holder at the end of the external tubing. Samples were collected from probes placed in the ileum and spiral colon at 0, 2, 4, 6, 8, 12, 24, 36, and 48 h post high-dose drug administration, and additionally 26, 28, 30, 32, 50, 52, 54, 56, 60, 72, 84, and 96 h after the first low-dose drug administration by changing the collection tubes at the predetermined time points. The collected fluid was aliquoted into cryogenic vials and immediately frozen at −80°C until assayed.

### Feces collection

Feces was collected directly from the rectum of each steer 0, 12, 24, 36, 48, 72, 96, 120, 144, and 168 h post single-dose drug administration, and additionally 60, 84, and 192 h after the first multiple-dose drug administration. Samples were placed into bags (Whirlpak®, Nasco, Fort Atkinson, WI) and stored briefly on ice before microbiological analysis. An aliquot of each sample was stored in a 2 ml cryogenic vial and frozen at −80°C. To avoid the residual effects of enrofloxacin on fecal bacteria between crossover arms of the study, only fecal samples collected from the first arm of each crossover study were analyzed and reported here.

### Determining active drug concentration

For plasma samples, we used solid-phase extraction, identical to the method in our previous papers (Davis et al., 2007). Plasma and tissue fluid samples were analyzed by reverse-phase high-pressure liquid chromatography with fluorescence (enrofloxacin and its metabolite ciprofloxacin) detection to determine the active concentrations of each drug as previously described (Davis et al., 2007; Foster et al., 2016). All drug concentrations were determined from calibration curves made from fortified blank plasma, intestinal and interstitial fluid collected from the experimental calves prior to antibiotic administration. Calibration curves were prepared from fortifying the blank matrix with reference drug standards of enrofloxacin and ciprofloxacin (United States Pharmacopeia, Rockville, MD) to validate the HPLC analysis and perform quality control assessments during the assay.

### Pharmacokinetic analysis

The drug concentrations were analyzed using standard pharmacokinetic methods to determine the drug disposition for each drug in each calf. A computer program (Phoenix, V. 6.1; Pharsight Corporation, Certara, St. Louis MO) was used to determine pharmacokinetic parameters as well as deriving statistical values.

Plasma, ISF, and intestinal drug concentrations were plotted on linear and semi-logarithmic graphs for analysis and for visual assessment of the best model for pharmacokinetic analysis. Specific models (e.g., one, two, etc. compartments) were determined for best fit based on visual analysis for goodness of fit and by visual inspection of residual plots. The best model fit was based on the equation described in the following formula:

$$\begin{array}{l} C=\frac{\;k_{01\;}\cdot \;D}{\text{V }\left(k_{01}-\;k_{10}\right)}\;\left(e^{-k_{10}\cdot t}-e^{{-k}_{01}\cdot t}\right) \end{array}$$

Where *C* is the plasma concentration, *t* is time, *k*_01_ is the non-IV absorption rate, assuming first-order absorption, *k*_10_ is the elimination rate constant, V is the apparent volume of distribution, and *D* is the non-IV dose. Secondary parameters calculated from the model included the peak concentration (C_MAX_), time to peak concentration (T_MAX_), area under the plasma-concentration vs. time profile (AUC), and the respective absorption and terminal half-lives (*t*½).

Data from some calves were analyzed using non-compartmental analysis (NCA) due to sparse sampling using the same pharmacokinetic program described above. For the NCA, the area under the plasma concentration vs. time curve (AUC) from time 0 to the last measured concentration (defined by the LOQ), was calculated using the log-linear trapezoidal method. The AUC from time 0 to infinity was calculated by adding the terminal portion of the curve, estimated from the relationship C_n_/λ_Z_, to the AUC_0__Cn_, where λ_Z_ is the terminal slope of the curve, and C_n_ is the last measured concentration point.

The relative drug transfer from the plasma compartment to the ISF and intestinal fluids was measured by calculation of a *penetration factor*. The penetration factor was determined by the ratio of AUC for the intestinal fluid to the AUC for plasma:

$$\begin{array}{l} Penetration\;Factor=\frac{AUC_{Intestinal\;Fluid\;or\;ISF}}{AUC_{Plasma}} \end{array}$$

### Quantification of *E. coli* from feces

One gram of feces was inoculated into 9 ml EC broth (Oxoid Ltd., Basingstoke, Hampshire, England) and vortexed. One ml was removed and serially diluted 10-fold in sterile phosphate-buffered saline, and 100 μl was plated in triplicate onto HardyCHROM™ ECC Media (Hardy Diagnostics, Santa Maria, CA). Plates were incubated overnight at 37°C and pink-violet colonies were counted to determine the CFU/ml of *E. coli*. Dilutions that yielded between 30 and 300 colonies on each of the three plates were counted to quantify *E. coli* based on the dilution. These three replicates at the counted dilution were averaged to determine the quantity of *E. coli* at each time point. The remaining enrichment was incubated overnight at 37°C, and if no growth was observed on direct plates, the samples were streaked for isolation on ECC plates and incubated overnight at 37°C. From the quantified or enrichment plate, eight colonies were randomly picked and streaked onto Columbia agar with 5% sheep blood (Remel, Lenexa, KS) and incubated overnight at 37°C. Following incubation, each isolate was transferred to a 2 ml cryogenic vial containing LB Broth (Sigma-Aldrich, St. Louis, MO) supplemented with 25% glycerol, vortexed, and frozen at −80°C.

### Determination of minimum inhibitory concentration

The minimum inhibitory concentration (MIC) of each *E. coli* isolate to enrofloxacin was determined using broth microdilution according to Clinical and Laboratory Standards Institute guidelines (Clinical Laboratory Standards Institute, 2013). Each isolate was grown overnight on blood agar. A single colony was inocluated into Mueller Hinton broth and brought to a 0.5 McFarland Standard. Fifty μl of bacterial suspension was inoculated into 50 μl of 2-fold serial dilutions of enrofloxacin ranging in concentration from 0.03 to 32 mg/L (USP, Rockville, MD). Plates were incubated overnight (18 h) at 37°C. The first well with no visible growth within a given isolate was determined to be the MIC.

### DNA extraction and 16S rRNA sequencing

Fresh feces collected at each time point were frozen in 1 gram aliquots for future analysis. From these samples, 50 mg of feces were extracted individually using a MoBio PowerMag Microbiome kit (Qiagen, Inc., Germanton, MD) optimized for the epMotion 5075 TMX (Eppendorf, Hauppauge, NY). The DNA libraries were prepared as described previously (Seekatz et al., 2015).

#### Illumina MiSeq sequencing of bacterial communities

The V4 region of the 16S rRNA gene was amplified from each sample using the Dual indexing sequencing strategy (Kozich et al., 2013). Sequencing was done on the Illumina MiSeq platform, using a MiSeq Reagent Kit V2 500 cycles (2 × 250 bp; Illumina cat# MS102-2003), according to the manufacturer's instructions with modifications (Kozich et al., 2013). Accuprime High Fidelity Taq (Life Technologies cat # 12346094) was used. PCR was performed using the conditions (Standard or Touch Down) shown by Seekatz (Seekatz et al., 2015). If additional template was used, the water volume was changed accordingly. PCR products were visualized using an E-Gel 96 with SYBR Safe DNA Gel Stain, 2% (Life technologies cat# G7208-02). Libraries were normalized using SequalPrep Normalization Plate Kit (Life technologies cat #A10510-01) following the manufacturer's protocol for sequential elution. The concentration of the pooled samples was determined using Kapa Biosystems Library Quantification kit for Illumina platforms (KapaBiosystems KK4824). The sizes of the amplicons in the library were determined using the Agilent Bioanalyzer High Sensitivity DNA analysis kit (cat# 5067-4626). The final library consisted of equal molar amounts from each of the plates, normalized to the pooled plate at the lowest concentration. Sequencing libraries were prepared according to Illumina's protocol for Preparing Libraries for Sequencing on the MiSeq (part# 15039740 Rev. D) for 2 or 4 nM libraries. If the library concentration was below 1 nM, an alternative method was used for denaturation (Quail et al., 2008). PhiX and genomes were added in 16S amplicon sequencing to create diversity. Sequencing reagents were prepared according to the Schloss SOP, and custom read 1, read 2 and index primers were added to the reagent cartridge. FASTQ files were generated for paired end reads.

### Microbiota analysis

The median number of sequences per group was 24,866. Analysis of the V4 region of the 16S rRNA gene was done using mothur (version 1.37.4; Schloss et al., 2009). The standard operating procedure at http://www.mothur.org/wiki/MiSeq_SOP was followed to process the MiSeq data. The paired-end reads were assembled into contigs and then aligned to the SILVA 16S rRNA sequence database (Pruesse et al., 2007; Quast et al., 2013) and classified to the family taxonomic level using the Wang method and an 80% bootstrap minimum (Wang et al., 2007) and the mothur-adapted RDP training set v9. Chimeric sequences were removed using UCHIME (Edgar et al., 2011). Sequences were clustered into operational taxonomic units (OTU) using a 3% sequence similarity threshold. The percentage relative abundance of bacterial Phyla and Family members in each sample was calculated. The inverse Simpson index on OTUs was used as a measure of alpha diversity.

Additional high-resolution analysis was performed on exact amplicon sequence variants (ASVs) generated by the DADA2 method (Callahan et al., 2016, 2017). ASV generation followed the DADA2 tutorial (https://benjjneb.github.io/dada2/tutorial.html), but with samples pooled for the ASV inference step in order to increase sensitivity to rare sequence variants. *E. coli* ASVs were identified by exact matching to sequenced *E. coli* genomes.

### Data analysis

Differences in the *E. coli* concentration between groups were determined by a one way ANOVA. ANOVA on ranks was used to compare median MIC over time within treatment groups. A *p*-value < 0.05 was considered significant. Differential abundance testing was performed using the DESeq2 package (Love et al., 2014), and following the recommendations for its application to microbiome sequencing data in McMurdie and Holmes (McMurdie and Holmes, 2014). Briefly, DESeq2 controls for the over-dispersion typical in microbiome sequencing data by fitting the abundance-variance relationship over all ASVs, and using that shrinkage estimator of the variance to test for significance. Testing for the treatment effect was performed on paired samples from each steer, before and after treatment, and after controlling for the steer. False discovery rate (FDR) was controlled using the Benjamini-Hochberg procedure (Benjamini and Hochberg, 1995).

### Deposition of data

Sequence data will be deposited in the SRA database upon acceptance of the manuscript.

## Results

### Pharmacokinetic modeling

**Figure 1:**
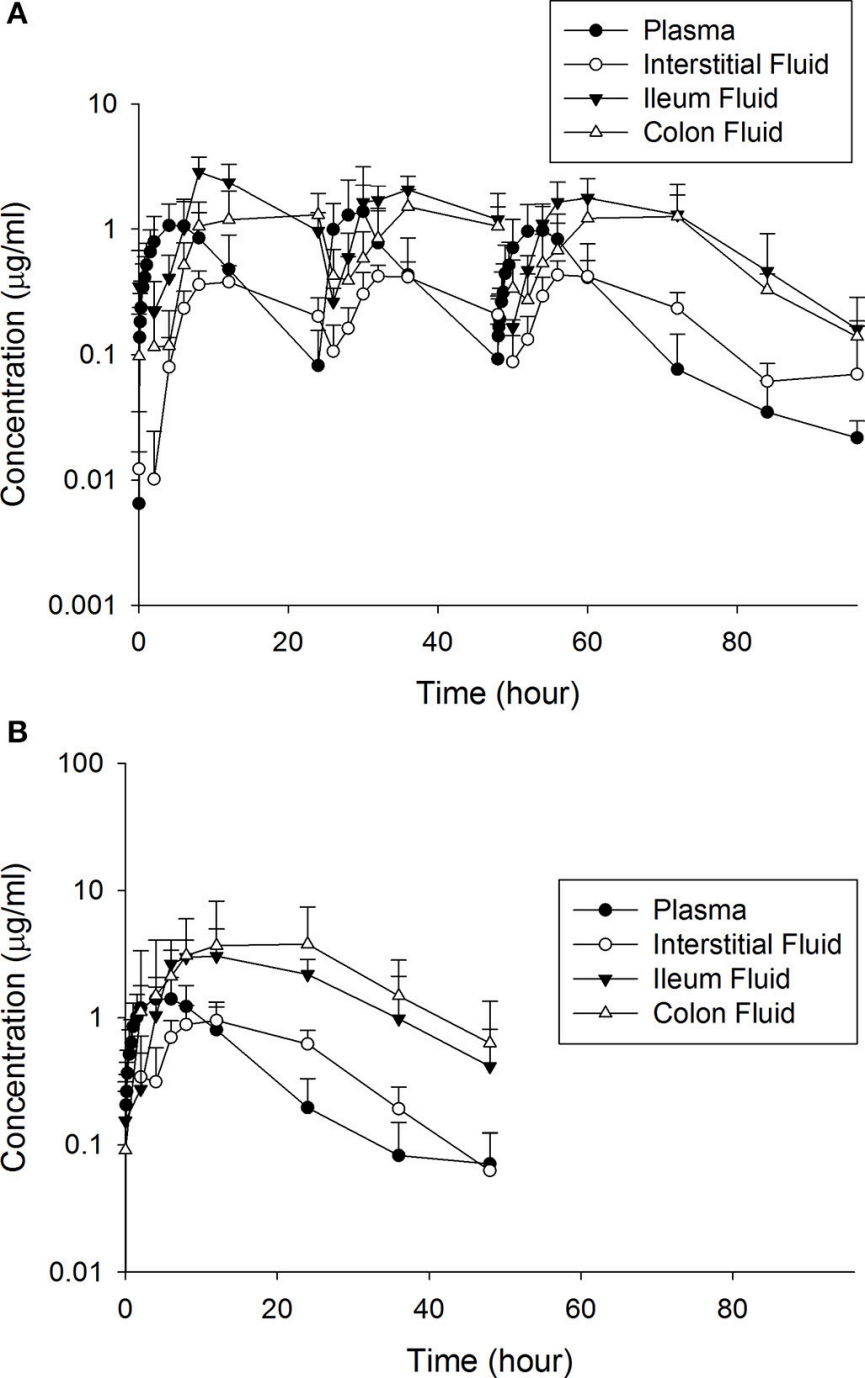
The combined concentrations of enrofloxacin and ciprofloxacin between the **(A)** multiple and **(B)** single dose treatment regimens.

Tables 1, 2 depict the results of the PK analysis for enrofloxacin and ciprofloxacin for the single and multiple dose regimens. The ciprofloxacin concentration accounted for 25.8% of total fluoroquinolone concentrations in the single dose regimen and 26.7% of total fluoroquinolone concentrations in the multiple dose regimen. Ciprofloxacin penetration from the plasma into the ISF was two times greater than enrofloxacin in the single dose regimen and four times greater than enrofloxacin in the multiple dose regimen. Enrofloxacin penetration into the ileum from the plasma (single dose = 491.86%, multiple dose = 451.03%) and into the colon from the plasma (single dose = 385.29%, multiple dose = 293.32%) exceeded the penetration into the ISF from the plasma (single dose = 96.03%, multiple dose = 49.57%). Figure 1 depicts the combined concentrations of enrofloxacin and ciprofloxacin over time with the two dosing regimens in plasma, interstitial fluid, ileum and spiral colon.

**Table 1:** Single dose results for enrofloxacin and ciprofloxacin.

	**Single dose study (12.5 mg/kg once)**							
		**Plasma**		**ISF**		**Ileum**		**Colon**	
**Parameter**	**Units**	**Mean**	**Std Dev**	**Mean**	**Std Dev**	**Mean**	**Std Dev**	**Mean**	**Std Dev**
**CIPROFLOXACIN**									
AUC (0 to Cn)	h^*^μg/mL	5.41	1.30	10.90	5.36	12.04	3.95	15.77	18.80
AUC (0 to infinity)	h^*^μg/mL	5.97	1.35	11.77	5.42	13.89	4.15	24.34	23.36
C_MAX_	μg/mL	0.36	0.15	0.49	0.30	0.55	0.30	0.70	0.75
Half-life	h	15.17	7.90	7.84	3.31	15.18	8.30	18.13	10.45
Elim Rate	1/h	0.06	0.03	0.10	0.03	0.06	0.04	0.05	0.02
MRT	h	18.23	6.83	21.25	4.35	27.67	9.35	33.40	15.95
Tmax	h	5.01	2.14	12.36	4.18	14.44	7.47	14.29	7.25
Penetration	%			204.34	82.79	233.15	93.61	267.02	329.13
**ENROFLOXACIN**									
AUC (0 to Cn)	h^*^μg/mL	16.27	8.78	11.15	3.61	71.84	34.97	80.60	71.55
AUC (0 to infinity)	h^*^μg/mL	16.74	9.12	10.97	3.37	79.76	46.44	118.99	91.88
C_MAX_	μg/mL	1.21	0.62	0.52	0.19	3.28	1.37	4.37	3.58
Half-life	H	9.70	6.01	7.36	4.04	13.74	7.83	12.29	6.82
Elim rate	1/h	0.10	0.05	0.11	0.04	0.06	0.03	0.07	0.03
MRT	h	11.43	2.91	18.66	5.66	23.02	6.06	24.81	12.17
T_MAX_	h	5.33	1.97	9.45	2.02	10.44	5.73	14.29	7.25
Penetration	%			96.03	61.24	491.86	284.88	385.29	281.40

* Penetration % = AUC tissue/AUC plasma.

*AUC, area-under-the-curve (plasma concentration vs. time curve) expressed as 0 to Cn (last time point), or to infinity; C_MAX_, peak concentration; Elim Rate, elimination rate; Half-life, terminal half-life (T$\frac{1}{2}$); MRT, mean residence time; TMAX, time of peak concentration; Penetration, extent of penetration (%) from plasma to tissue compartment*.

**Table 2:** Multiple dose results for enrofloxacin and ciprofloxacin.

	**Multiple dose study (5 mg/kg** × **3)**							
		**Plasma**		**ISF**		**Ileum**		**Colon**	
**Parameter**	**Units**	**Mean**	**Std Dev**	**Mean**	**Std Dev**	**Mean**	**Std Dev**	**Mean**	**Std Dev**
**CIPROFLOXACIN**									
Accumulation Index	(blank)	1.26	0.37	1.08	0.03	1.46	0.26	1.29	0.28
AUC tau	h^*^μg/mL	2.99	1.41	3.78	1.01	8.02	2.10	3.85	3.18
AUC (0 to Cn)	h^*^μg/mL	3.24	1.62	5.29	1.37	10.14	4.36	4.71	3.02
AUC (0 to infinity)	h^*^μg/mL	3.35	1.65	5.36	1.44	13.85	5.05	4.81	3.10
C_MAX_	μg/mL	0.25	0.10	0.21	0.06	0.52	0.14	0.31	0.24
Half-life	h	9.82	7.24	6.25	0.97	14.13	5.08	10.41	6.25
Elim Rate	1/h	0.10	0.05	0.11	0.02	0.06	0.03	0.10	0.08
MRT	h	11.37	1.99	21.66	3.43	26.15	8.97	27.36	11.46
Tmax	h	6.00	1.55	11.64	5.50	6.00	4.90	13.33	9.38
Penetration	%	.	.	196.52	98.74	340.48	143.06	186.85	162.56
**ENROFLOXACIN**									
Accumulation Index	(blank)	1.08	0.08	1.12	0.12	1.12	0.13	1.25	0.23
AUC tau	h^*^μg/mL	8.57	6.48	3.17	2.14	24.27	9.49	19.09	10.51
AUC (0 to Cn)	h^*^μg/mL	8.97	6.98	4.10	2.68	30.68	19.12	24.24	17.00
AUC (0 to infinity)	h^*^μg/mL	9.08	7.01	4.30	2.77	37.05	17.80	34.79	16.01
C_MAX_	μg/mL	0.84	0.54	0.20	0.14	1.66	0.67	1.11	0.59
Half-life	h	5.78	2.56	7.07	2.79	7.05	2.94	9.97	4.49
Elim Rate	1/h	0.15	0.08	0.11	0.03	0.11	0.03	0.08	0.03
MRT	h	8.46	1.34	19.12	2.66	20.70	5.97	31.94	5.69
T_MAX_	h	4.55	1.57	12.00	4.38	14.67	7.45	19.50	6.21
Penetration	%			49.57	12.79	451.03	179.46	283.32	171.96

* Penetration % = AUC tissue/AUC plasma.

*AUC, area-under-the-curve (plasma concentration vs. time curve) expressed as 0 to Cn (last time point), or to infinity; C_MAX_, peak concentration; Elim Rate, elimination rate; Half-life, terminal half-life (T$\frac{1}{2}$); MRT, mean residence time; TMAX, time of peak concentration; Penetration, extent of penetration (%) from plasma to tissue compartment*.

### Concentration of *E. coli*

**Figure 2:**
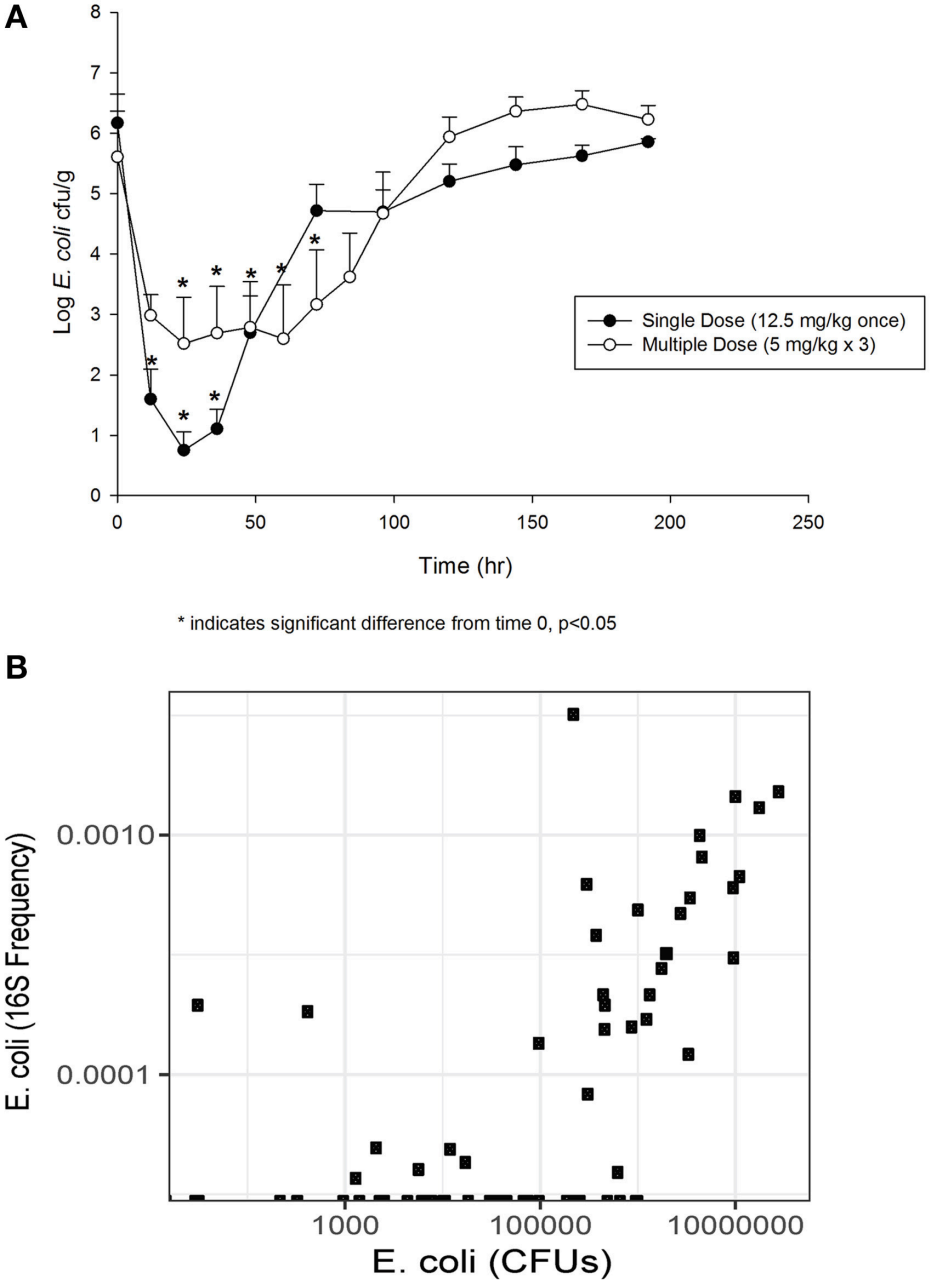
**(A)** Fecal *E. coli* concentration over time after treatment with either a single dose (12.5 mg/kg once) or multiple dose (5 mg/kg once a day for 3 days) of enrofloxacin. Mean ± SD. ^*^ indicates a significant difference from time 0, *p* < 0.05. **(B)** Comparison of culturable *E. coli* concentration and the frequency of the *E. coli* 16S sequence variant.

The concentration of *E. coli* recovered at 12 (1.60 log_10_ CFU/g), 24 (0.75 log_10_ CFU/g) and 36 (1.11 log_10_ CFU/g) h was significantly different from 0 h (6.17 log_10_ CFU/g) in the single dose group (Figure 2A). By 72 h post-treatment, the *E. coli* concentration (4.72 log_10_ CFU/g) had returned to baseline. In animals administered the multiple dose regimen, the concentrations at 24, 36, 48, 60 and 72 h were significantly different from 0 h (Figure 2A), and then returned to baseline by 96 h post-treatment. The correlation between culturable and sequenced *E. coli* concentrations was 0.557 over all samples, increasing to 0.808 when considering only those samples with >500,000 CFUs/g, which roughly corresponds to the minimum detectable frequency of 0.0001 for the 16S sequencing in this study (~10 k reads per sample; Figure 2B). This correlation suggests that the culture results agree with the sequencing findings presented below.

### *E. coli* minimum inhibitory concentration

**Figure 3:**
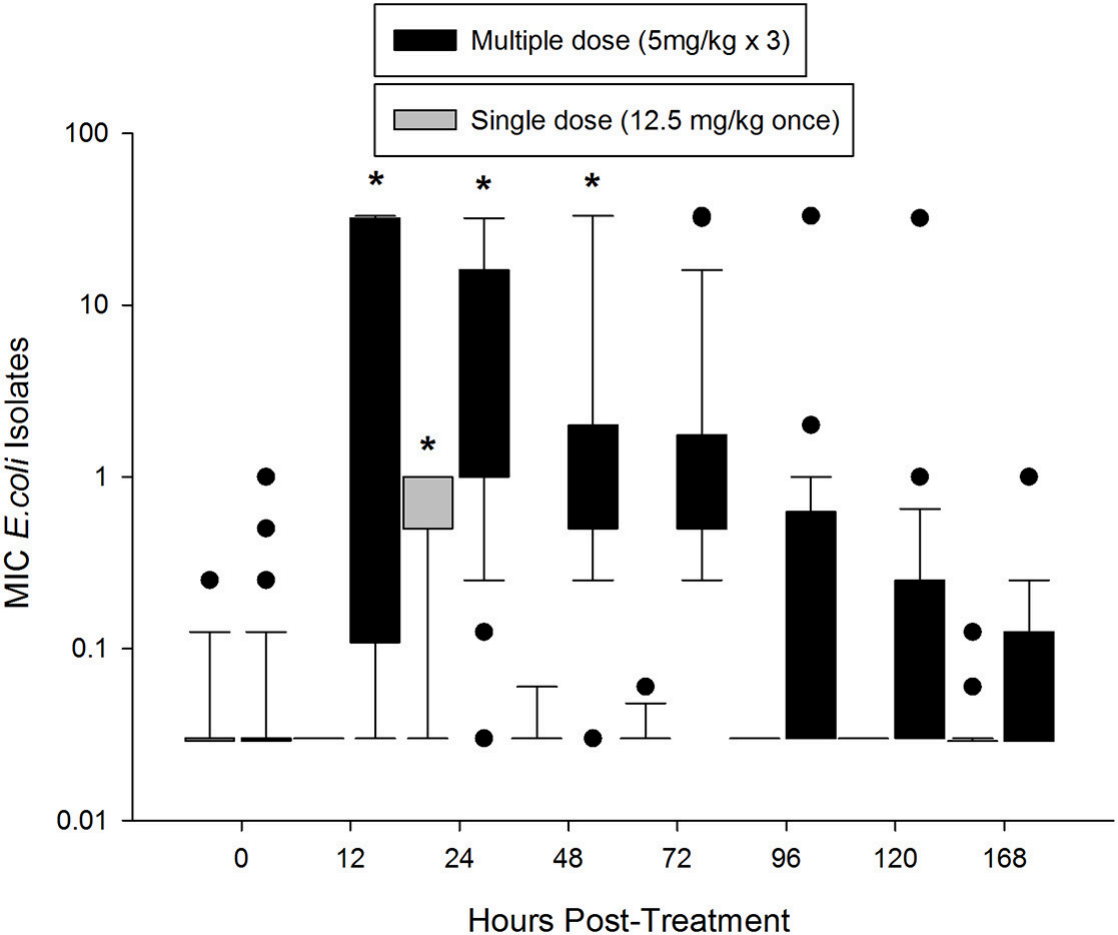
Median MIC of enrofloxacin in fecal *E. coli* isolates over time after treatment with either a single dose (12.5 mg/kg once) or multiple dose (5 mg/kg once a day for 3 days) of enrofloxacin. ^*^ indicates a significant difference from time 0, *p* < 0.05.

Prior to treatment, 84% *E. coli* isolates had an MIC of 0.03 mg/L or less, which is below the epidemiological cutoff value of 0.12 mg/L (Figure 3). The median MIC (0.029 mg/L) was not different between the two groups at time 0. In the single dose group, the median MIC significantly increased at 24 h post-treatment (0.5 mg/L), but returned to baseline by 48 h (0.03 mg/L). The median MIC of *E. coli* isolates from the multiple dose group significantly increased by 12 h after treatment (0.75 mg/L), and remained increased through 48 h (1.0 mg/L; Figure 3). When comparing individual time points across treatment regimens, the median MIC of *E. coli* isolates from the multiple dose group was significantly higher than the single dose group starting at 12 h and remained higher through the end of the study (Figure 3).

### Alterations in the fecal microbiota

**Figure 4:**
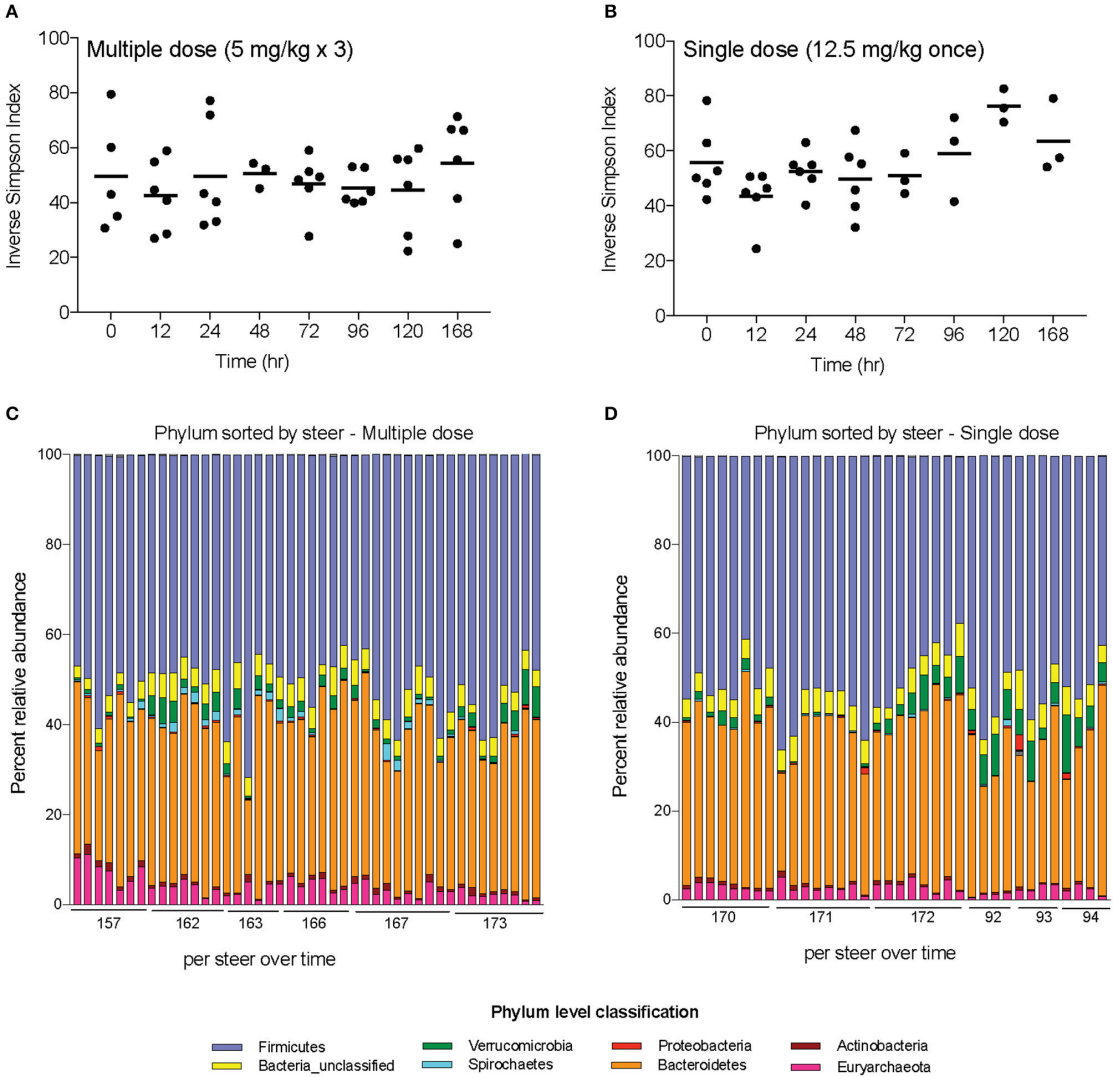
Changes in diversity and bacterial community membership in steers before, during, and after treatment with enrofloxacin. Alpha-diversity measurements in steers treated with **(A)** multiple and **(B)** single doses of antibiotics. Bar plots depict the mean percent abundances of the top bacterial Phyla in steers treated with **(C)** multiple and **(D)** single doses of antibiotics.

**Figure 5:**
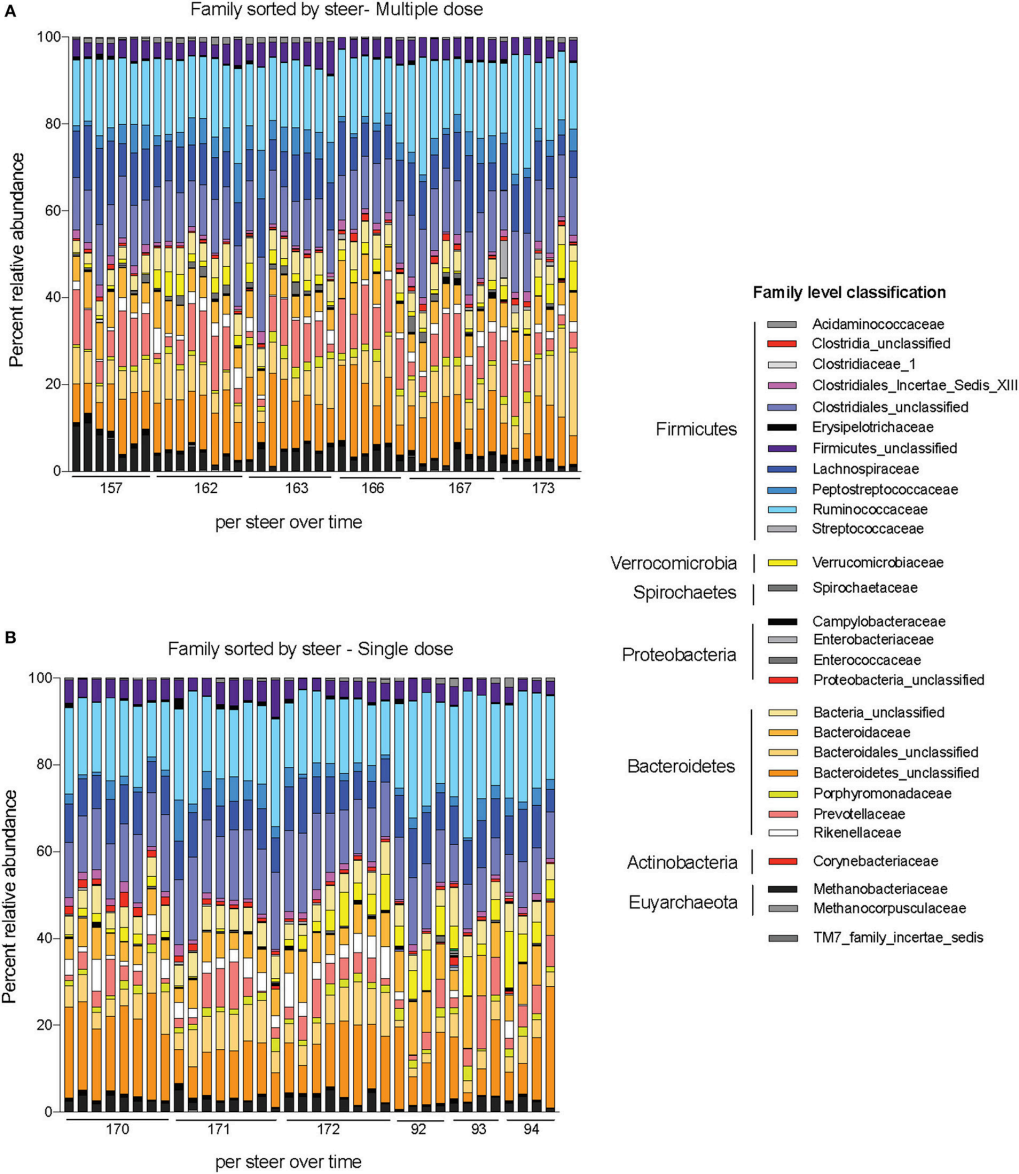
Changes to bacterial community membership in steers before, during, and after treatment with enrofloxacin. Bar plots depict the mean percent abundances of the top bacterial Families in steers treated with **(A)** multiple and **(B)** single doses of antibiotics.

Alpha-diversity, a measure of the diversity of bacterial phyla within each sample, showed no significant change over time in either dosing regimen (Figures 4A,B). There were subtle changes to the fecal microbial community at the Phylum (Figures 4C,D), and Family (Figures 5A,B) level. One ASV from the Family Prevotellaceae significantly increased in frequency at 48 h, while several ASVs from the Rikenellaceae, Christensenellaceae, Erysipelotrichaceae, and Enterobacteriaceae Families significantly decreased in frequency at a FDR of 0.01 (Table 3). At 168 h, only one ASV from the Family Erysipelotrichaceae, Genus Faecalitalea that was differentially abundant even at a relaxed FDR threshold of 0.25 (data not shown).

**Table 3:** The relative abundances of each 16S rRNA gene sequence variant at 0 h (before treatment) and at 48 h (after treatment) were compiled for each steer.

**Base mean**	**log2 Fold change**	***p*-value**	**padj**	**Phylum**	**Class**	**Order**	**Family**	**Genus**	**Sequence**
625.4	4.10	8.93E-08	2.38E-05	Bacteroidetes	Bacteroidia	Bacteroidales	Prevotellaceae	Prevotellaceae_UCG-003	TACGGAAGAT GCGAGCGTTA TCCGGATTTA TTGGGTTTAA AGGGAGCGTA GGCGGGCTGT TAAGTCAGCG GTAAAATGTC AAGGCCCAAC CTTGTCCTGC CGTTGAAACT GGCGGTCTTG AATGCACACA AGGGAGATGG AATTCGTCGT GTAGCGGTGA AATGCTTAGA TATGACGAAG AACTCCGATT GCGAAGGCAG TCTCCTGGGG TGTAATTGAC GCTGAGGCTC GAAAGTGCGG GTATCAAACA GG
34.9	−5.73	1.32E-06	0.000132	Bacteroidetes	Bacteroidia	Bacteroidales	Rikenellaceae	Alistipes	TACGGAGGAT CCAAGCGTTA TCCGGATTTA TTGGGTTTAA AGGGTGCGTA GGCGGTTTGA TAAGTTAGAG GTTAAATGTC AGTGCTCAAC ACTGGCCTGC CTCTAATACT GTTGGACTAG AGAGTAGATG CGGTAGGCGG AATGTATGGT GTAGCGGTGA AATGCGTAGA GATCATACAG AACACCGATT GCGAAGGCAG CTTACCAAAC TATATCTGAC GTTGAGGCAC GAAAGCGTGG GTAGCAAACA GG
17.26	−6.21	1.65E-07	3.29E-05	Bacteroidetes	Bacteroidia	Bacteroidales	Rikenellaceae	Alistipes	TACGGAGGAT CCAAGCGTTA TCCGGATTTA TTGGGTTTAA AGGGTGCGTA GGCGGTTTTA TAAGTTAGAG GTTAAATGTC AGGGCTCAAC TCTGGCCTGC CTCTAATACT GTAGGACTAG AGAGTAGATG CGGTAGGCGG AATGTATGGT GTAGCGGTGA AATGCGTAGA GATCATACAG AACACCGATT GCGAAGGCAG CTTACCAAAC TATATCTGAC GTTGAGGCAC GAAAGCGTGG GTAGCAAACA GG
25.4	−2.37	9.79E-05	0.006527	Firmicutes	Clostridia	Clostridiales	Christensenellaceae	Christensenell aceae_R-7_group	TACGTAGGGG GCGAGCGTTG TCCGGAATGA TTGGGCGTAA AGGGCGCGTA GGCGGCCTGG TAAGTCTGGA GTGAAAGTCC TGCTTTCAAG GTGGGAATTG CTTTGGATAC TGCTAGGCTC GAGTGCAGGA GAGGAAAGCG GAATTACCGG TGTAGCGGTG AAATGCGTAG AGATCGGTAG GAACACCAGT GGCGAAGGCG GCTTTCTGGA CTGAAACTGA CGCTGAGGCG CGAAAGCGTG GGGAGCAAA CAGG
12.7	−5.88	3.77E-07	5.02E-05	Bacteroidetes	Bacteroidia	Bacteroidales	Rikenellaceae	Alistipes	TACGGAGGAT CCAAGCGTTA TCCGGATTTA TTGGGTTTAA AGGGTGCGTA GGCGGTTTGG TAAGTTAGAG GTGAAATTTC AGGGCTCAAC CTTGACATTG CCTCTGATAC TGCCGAGCTA GAGAGTAGTT GCTGTGGGCG GAATGTATGG TGTAGCGGTG AAATGCTTAG AGATCATACA GAACACCGAT TGCGAAGGCA GCTCACAAAA CTATATCTGA CGTTGAGGCA CGAAAGCGTG GGTAGCAAAC AGG
13.0	−3.64	8.99E-06	0.00072	Firmicutes	Clostridia	Clostridiales	Christensenellaceae	Christensenell aceae_R-7_group	TACGTAGGGG GCAAGCGTTG TCCGGAATGA CTGGGCGTAA AGGGCGTGTA GGCGGCTTTT TAAGTGTGAA GTGAAAGTCC TGCTTTCAAG GTGGGAATTG CTTTGCAAAC TGGAGAGCTT GAGTGCGGAA GAGGTAAGTG GAATTCCCAG TGTAGCGGTG AAATGCGTAG AGATTGGGAG GAACACCAGT GGCGAAGGCG ACTTACTGGG CCGCAACTGA CGCTGAGGCG CGAAAGCGTG GGGAGCGAAC AGG
11.8	−3.53	1.52E-06	0.000135	Bacteroidetes	Bacteroidia	Bacteroidales	Rikenellaceae	Rikenellace ae_RC9_gut_group	TACGGGGGAT GCAAGCGTTA TCCGGATTTA TTGGGTTTAA AGGGTGCGTA GGCTGTCCGG TAAGTCAGCG GTGAAATTTA GGGGCTCAAC CTCTACCGTG CCGTTGATAC TGTCGGGCTA GAATGCGGAT GCCGTGGGAG GAATGTGTGG TGTAGCGGTG AAATGCATAG ATATCACACA GAACACCGAT TGCGAAGGCA TCTCACGAAT CCGCAATTGA CGCTGATGCA CGAAAGCGTG GGGATCAAAC AGG
20.8	−5.91	2.73E-09	2.19E-06	Firmicutes	Erysipelotrichia	Erysipelotrichales	Erysipelotrichaceae	Faecalitalea	TACGTAGGTG GCGAGCGTTA TCCGGAATCA TTGGGCGTAA AGGGTGCGTA GGTGGCAGAA TAAGTCTGAA GTAAAAGGCT GCAGCTCAAC TGCAGTATGC TTTGGAAACT GTTCAGCTAG AGTGCGGAAG AGGGCGATGG AATTCCATGT GTAGCGGTAA AATGCGTAGA TATATGGAGG AACACCAGTG GCGAAGGCGG TCGCCTGGTC CGTAACTGAC ACTGAGGCAC GAAAGCGTGG GGAGCAAATA GG
12.9	−4.43	2.96E-07	4.73E-05	Firmicutes	Clostridia	Clostridiales	Christensenellaceae	Christensenellaceae_R-7_group	TACGTAGGGG GCAAGCGTTG TCCGGAATGA CTGGGCGTAA AGGGCGTGTA GGCGGCTCTT TAAGTCTGAA GTGAAAGTCC TGCTTTCAAG GTGGGAATTG CTTTGGAGAC TGGAGAGCTT GAGTGCGGAA GAGGTAAGTG GAATTCCCAG TGTAGCGGTG AAATGCGTAG AGATTGGGAG GAACACCAGT GGCGAAGGCG ACTTACTGGG CCGTAACTGA CGCTGAGGCG CGAAAGCGTG GGGAGCGAAC AGG
11.9	−3.84	2.08E-08	8.31E-06	Bacteroidetes	Bacteroidia	Bacteroidales	Rikenellaceae	dgA-11_gut_group	TACGGAGGAT GCGAGCGTTA TCCGGATTTA TTGGGTTTAA AGGGTGCGTA GGCGGTTACT GTAAGTCAGT GGTGAAATTT TGATGCTTAA CATTAAAAGT GCCATAGATA CTGCAGAGCT GGAATGGGGA TGCTGTCAGC GGAATGTGTA GTGTAGCGGT GAAATGCATA GATATTACAC AGAACACCGA TTGCGAAGGC AGCTGACAAA TCCTTTATTG ACGCTGATGC ACGAAAGTGT GGGGATCAAA CAGG
9.4	−4.85	9.11E-07	0.000104	Cyanobacteria	Melainabacteria	Gastranaerophilales	NA	NA	TACGGGGGAT GCAAGCGTTG TCCGGAATCA TTGGGCGTAA AGAGTTCGTA GGTGGCCTGT TAAGTCTGGT GTTAAATGCA GAGGCTCAAC TTCTGTTCGG CACTGGATAC TGGCAAGCTT GAATGCGGTA GAGGTAAAGG GAATTCCTGG TGTAGCGGTG AAATGCGTAG ATATCAGGAG GAACATCGGT GGCGAAAGCG CTTTACTGGG CCGTAATTGA CACTGAGGAA CGAAAGCCAG GGTAGCAAAT GGG
4.72	−5.03	4.84E-05	0.00352	Proteobacteria	Gammaproteo bacteria	Enterobacteriales	Enterobacteriaceae	Escherichia/Shigella	TACGGAGGGT GCAAGCGTTA ATCGGAATTA CTGGGCGTAA AGCGCACGCA GGCGGTTTGT TAAGTCAGAT GTGAAATCCC CGGGCTCAAC CTGGGAACTG CATCTGATAC TGGCAAGCTT GAGTCTCGTA GAGGGGGGTA GAATTCCAGG TGTAGCGGTG AAATGCGTAG AGATCTGGAG GAATACCGGT GGCGAAGGCG GCCCCCTGGA CGAAGACTGA CGCTCAGGTG CGAAAGCGTG GGGAGCAAAC AGG

*The DESeq2 method was used to test for significant changes in relative abundance, while controlling for the sampled steer. Sequence variants that were significant when controlling for an FDR of 0.01 using the Benjamini-Hochberg method are reported. The log2FoldChange indicates the fold change from 0 to 48 h and the baseMean indicates the average starting abundance at 0 h*.

## Discussion

Enrofloxacin is commonly used for treatment of bovine respiratory disease (National Animal Health Monitoring System, 2011), but little is known about the relative impact of the two dosing regimens on AMR in fecal bacteria. In this study, subcutaneous administration of this fluoroquinolone at either dose resulted in high concentrations of active drug in the ileum and colon. Ciprofloxacin penetrates the ISF to a much larger extent than enrofloxacin. Our earlier studies showed that lower protein binding for ciprofloxacin (19 ± 5 vs. 46 ± 4%) might account for this difference in diffusion from plasma to interstitial fluid (Davis et al., 2007). However, this difference in protein binding does not appear to have the same effect on the penetration of enrofloxacin from the plasma to the intestine as enrofloxacin penetration was 450–490% into the ileum and 280–380% into the colon, similar to our previous study (Foster et al., 2016). This suggests that there may be a transporter protein or biliary excretion responsible for the high penetration of these fluoroquinolones into the intestine relative to both the plasma and ISF. Active secretion of fluoroquinolones into the intestine (Griffiths et al., 1993, 1994; Dautrey et al., 1999) and other tissues (Pulido et al., 2006; Alvarez et al., 2008; Mulgaonkar et al., 2013) has been demonstrated in other studies. Further, the difference in the AUC in the colon was greater than would be expected based on the different doses, indicating that this transport mechanism may be somewhat concentration dependent.

The concentration of *E. coli* shows an inverse relationship with the concentration of enrofloxacin, as the single dose quickly lowered the *E. coli* concentration by 5 log_10_ CFU/g of feces. By 96 h post-treatment, *E. coli* fecal concentrations returned back to baseline for both the single and multiple dose regimens. This precipitous decline and rapid re-establishment of *E. coli* concentrations with low MIC values toward enrofloxacin is consistent with the “inverted U” concept to describe the effects of fluoroquinolone concentration on bacterial resistance (Tam et al., 2007). With high fluoroquinolone exposure, as we observed in the intestine of these cattle, *E. coli* were significantly, but temporarily suppressed (Figure 2A). By 96 h after treatment, the median MIC of the isolates from the single dose regimen reverted to the MIC of a wild-type population as the *E. coli* concentration rebounded. The return to baseline concentration and MIC corresponded with elimination of the drug from the intestine. In the multiple dose group, the median MIC increased to a greater degree than in the single dose group. Again, the change we observed in MIC was temporary, suggesting a lack of fitness for bacteria with a high MIC in the intestine of these cattle. This time for return to a wild-type MIC range is well within the slaughter withdrawal time of 28 days (Weatherbee, 2012) established by the FDA to prevent drug residues. Our results indicates that when FDA-approved slaughter withdrawal times are observed for enrofloxacin, it may mitigate the risk of transfer of resistant *E. coli* isolates through the food supply at slaughter. As we only assessed the MIC of *E. coli* in this study, it is unclear if these findings can be extrapolated to other enteric bacteria.

While evaluating changes to the complete fecal microbiome, we demonstrated only minor changes in community structure after treatment, and there was no change in microbial diversity. The transient changes that were seen were primarily in bacteria predominantly associated with the rumen microbiota (Prevotellaceae, Rikenellaceae, Christensenellaceae, and Erysipelotrichaceae; Mao et al., 2015). Further, the relatively small, short-lived changes found in steers treated with enrofloxacin is in contrast to findings in chickens (Li et al., 2017), mice (Choo et al., 2017), and humans (Stewardson et al., 2015; Pop et al., 2016) administered a fluoroquinolone that demonstrate significant changes at the phylum and genus levels which persist for a week or more. Potentially, the large microbial population of the rumen provides a source of microbes to continually repopulate the intestinal environment, leading to minimal changes in the fecal microbiota in ruminants compared to simple-stomached animals. A similar approach to measure active drug concentrations in the rumen and assess the impact of parenteral drugs on the rumen microbiome would be valuable to address this question. Alternatively, differences in the dosing regimens used in these studies compared those used in cattle may also influence these findings as each of these studies administered the drugs for longer durations and by a different route.

The study was conducted in healthy Holstein steers, and the PK of enrofloxacin or associated microbiological changes may not represent that of sick calves. Nonetheless, our results are similar to findings in the National Antimicrobial Monitoring System that suggest resistance to fluoroquinolones in bovine *E. coli* isolates is rare in cattle at slaughter (Food and Drug Administration, 2018). The PK findings and short duration of changes to fecal bacteria found in this study may help explain the rare incidence of fluoroquinolone-resistant *E. coli* isolates in cattle at slaughter (1.2% of isolates in beef cattle in 2015). Resistance to fluoroquinolones in *Salmonella* isolates was similarly uncommon in beef cattle at slaughter, while rates of fluoroquinolone-resistance in *Enterococcus* spp. and *Campylobacter* spp. tended to be higher. As we only examined the impact on fecal *E. coli*, extrapolation of these findings to other fecal bacteria may not be appropriate, and warrants additional investigation.

In conclusion, enrofloxacin penetrates the ileum and colon of cattle at high concentrations with either the single or multiple dosing regimens causing a significant reduction in fecal *E. coli* concentration that resolved within 4 days of treatment. With the multiple dose regimen, there was a small increase in median MIC. Yet, the median MIC returned to baseline by 2 weeks post-treatment. Assessment of the microbiome after treatment did not show clinically significant changes with either dosing regimen. From this study, it appears that dosing regimens in which antimicrobial concentrations rapidly achieve pharmacodynamic targets and are then quickly eliminated from the GI tract will minimize the effect on the fecal microbiota of cattle.

## Author contributions

MJ, CT, MP, and DF contributed to study design, data collection and analysis, and manuscript preparation. KF, BC, and TP contributed to data collection and analysis, and manuscript preparation.

### Conflict of interest statement

DF, MJ, and MP have received research funding from and consulted for Bayer Animal Health. The remaining authors declare that the research was conducted in the absence of any commercial or financial relationships that could be construed as a potential conflict of interest.
